# Graph Attention Interaction Aggregation Network for Click-Through Rate Prediction

**DOI:** 10.3390/s22249691

**Published:** 2022-12-10

**Authors:** Wei Zhang, Zhaobin Kang, Lingling Song, Kaiyuan Qu

**Affiliations:** Department of Artificial Intelligence Education, Central China Normal University, Wuhan 430079, China

**Keywords:** click-through rate prediction, recommender systems, feature interaction, graph neural network, attention mechanism

## Abstract

Click-through rate prediction is a critical task for computational advertising and recommendation systems, where the key challenge is to model feature interactions between different feature domains. At present, the main click-through rate prediction models model feature interactions in an implicit way, which leads to poor interpretation of the model, and the interaction between each pair of features may introduce noise into the model, thus limiting the predictive ability of the model. In response to the above problems, this paper proposes a click-through rate prediction model (GAIAN) based on the graph attention interactive aggregation network, which explicitly obtains cross features on the graph structure. Our specific method is to design a feature interactive selection mechanism to select cross features that are beneficial to model prediction, reducing model noise and reducing the risk of model overfitting. On this basis, the bilinear interaction function is integrated into the aggregation strategy of the graph neural network, and the fine-grained intersection features are extracted in a flexible and explicit way, which makes graph neural networks more suitable for modeling feature interactions and enhances the interpretability of the model. Compared with several other state-of-the-art models on the Criteo and Avazu datasets, the experimental results show the superiority of the model.

## 1. Introduction

Click-through Rate (CTR) prediction plays a central role in the field of computational advertising and recommendation systems. In this field, whether an item (such as a movie or an advertisement) is recommended or not is usually affected by the probability that a user will click on it, and CTR can estimate the probability that a user will click on a given item [1]. In an online advertising application, the ranking strategy for candidate ads is usually by CTR × bid, where bid is the profit the system receives after the ad is clicked. In such applications, the performance of the CTR prediction model is one of the core factors to determine the benefits of the system [2].

The key challenge in the CTR prediction task is to model feature interactions effectively. However, most of the features in recommender systems are high-dimensional and sparse categorical features, and it is important to consider their interactions when using these features for CTR prediction. For example, it is reasonable to recommend cosmetics to a 20-year-old female. In this case, the third-order combined feature <Gender = Female, Age = 20, Product Category = Cosmetics> is very useful information for prediction. Factorization Machine (FM) [3,4] is an efficient and classical method for modeling feature interactions. The core idea of FM is to explicitly model second-order feature interactions by parameterizing the weights of the crossed features as the inner product of the embedding vectors of the original features. Due to its simplicity and effectiveness, FM has been widely used in recommender systems and CTR prediction tasks [5,6]. However, although FM takes into account all feature interactions, it suffers from two major drawbacks. First, it is inappropriate to consider interactions among all features, as interactions among some features may be detrimental to prediction. Modeling these useless feature interactions will introduce noise to the model and lead to overfitting [7] Second, FM cannot model higher-order feature interactions. Although there have been some efforts to extend FM to higher order, such as HOFM [8] it has high complexity in practical application.

In order to solve the problem that FM lacks to distinguish the importance of feature interaction, Attentional Factorization Machines (AFM) [9] use an attention mechanism to learn the weight of each feature interaction, but it also cannot model high-order feature interaction. In response to the problem that FM cannot model high-order feature interactions, many CTR prediction models based on deep neural networks (DNNs) have been proposed to learn high-order feature interactions. For example, Factorization-Machine Supported Neural Networks (FNN) [10] uses FM to pre-train the input features to obtain second-order combined features and then applies DNN to model higher-order feature interactions. Similarly, models such as DeepFM [6], NFM [11] and DeepCrossing [12] utilize deep neural networks to model higher-order feature interactions. However, these DNN-based models all learn high-order feature interactions in an implicit manner, thus lacking good model interpretability.

Graph neural network (GNN) is a class of models used to learn the representation of nodes in graphs. It has achieved remarkable success in different graph learning tasks. Most popular GNN models follow a neighborhood aggregation scheme to learn node representations through message passing between local neighbor nodes in the graph. Therefore, higher-order interactions between nodes can be explicitly encoded into node embeddings by stacking layer numbers. Fi-GNN [13] first proposed to apply graph neural network to solve the problem of high-order feature interaction. It represented multi-domain features into a fully connected graph and then used a gated graph neural network (GGNN) [14] to model the feature interaction on the feature graph. However, on the one hand, not all feature interactions are beneficial, and on the other hand, GNNs are based on the assumption that neighbors share similar features [15], which does not conform to the essence of feature interaction modeling.

Aiming at the shortcomings of previous models, and in order to give full play to the advantages of GNN in explicitly modeling higher-order feature interactions, this paper proposes a click-through rate prediction model(GAIAN) based on graph attention interaction aggregation network.

In summary, the contributions of this paper are as follows:

- In order to solve the problems existing in previous models in feature interaction modeling, a CTR prediction model(GAIAN) based on graph attention interaction aggregation network was proposed to model feature interaction;
- A feature interaction selection mechanism is designed, which reduces model noise and reduces the risk of model overfitting by selecting cross-features that are beneficial to model prediction;
- A bilinear interaction function is integrated into the aggregation strategy of graph neural networks to extract fine-grained intersection features in a flexible and explicit manner.

The rest of this paper is organized as follows. In Section 2, we review related works that are relevant to our proposed model, followed by introducing our proposed model in Section 3. In addition, we give an experimental comparison and analysis with other models in Section 4. Finally, we summarize this work in Section 5.

## 2. Related Works

In this section, the work related to this paper will be briefly reviewed from three aspects.

### 2.1. Feature Interaction Modeling

Feature interaction modeling is a key task in CTR prediction, so it has been widely studied. To explicitly model feature interactions, many factorization machine based methods have been proposed for high-dimensional data, such as Factorization Machine (FM) [4], Field-aware Factorization Machine (FFM) [5], Field-weighted Factorization Machine (FwFM) [16,17] and Field matrix Factorization Machine (FmFM) [18]. In addition, there are several works aimed at learning weights for different feature interactions, including Attentional Factorization Machine (AFM) [9], Dual-attentional Factorization Machine (DFM) [19], Dual Input-aware Factorization Machines (DIFM) [20]. However, these methods do not explore the impact of higher-order feature interactions on predictions.

Due to the increasing number of samples and the dimension of features, many deep learning-based models have been proposed to learn higher-order feature interactions. The Wide & Deep [21] model jointly trains a Wide model and a Deep model, where the Wide model exploits the effectiveness of feature engineering, and the Deep model learns high-order feature interactions. While the Wide part is useful, feature engineering is expensive and requires people with domain knowledge. In order to avoid feature engineering, DeepFM [6] introduced FM as a Wide part to simulate low-order feature interactions, and used DNN to learn higher-order feature interactions. Different from DeepFM, PNN [1] designs a product layer for second-order feature interaction and then concatenates the original features into DNN, and obtains similar performance. However, all of these approaches utilizing DNNS learn higher-order feature interactions in an implicit manner, and there is currently a lack of interpretation of the feature interaction modeling process and model results. Therefore, some researchers try to use the attention mechanism to model feature interactions in an interpretable way. The AutoInt [22] model uses residual connections based on the multi-head attention mechanism and explicitly realizes feature interactions of different orders by stacking multiple multi-head attention modules. The InterHAT [23] model replaces the multi-head attention mechanism with the Transformer [24] structure on the basis of the AutoInt model, which further improves the efficiency of model training. However, although these methods utilize attention mechanisms to account for the weight of each pair of feature interactions, fully aggregating these interactions still introduces noise and reduces prediction accuracy.

To address these issues, some works attempt to automatically extract useful feature interactions. For example, AutoFIS [25] searches and models beneficial feature interactions through a gating mechanism and then inputs them to the FM model. However, there is a loss of information between the two phases and the modeling process remains unexplained.

### 2.2. Graph Neural Network

A graph structure consists of nodes and edges, where edges represent relationships between nodes. GNN [26] is a method to operate graph structure data based on deep learning. Most popular GNN models follow the neighborhood aggregation strategy, and different aggregation schemes are proposed to learn the representation of nodes by aggregating the features of neighboring nodes layer by layer. Therefore, higher-order relationships between nodes can be explicitly modeled by stacking the number of layers. GCN [27] obtains the information of neighbor nodes by using convolutional aggregators to operate the first-order neighborhood around each node. GraphSAGE [28] designed three different message aggregators, mean/LSTM/pooling, to aggregate neighbor messages. GAT [29] aggregates the information of neighbor nodes by using attention mechanism to assign different weights to different neighbors. Furthermore, Cross-GCN [30] introduces a cross feature operator to explicitly model cross features of arbitrary order features.

In recent years, GNNs have been widely used in the field of recommender systems [31]. GIN [32] exploits user behavior to construct a co-occurring item graph to mine user intent. GCMC [33] treats the recommendation task as a link prediction problem and uses a graph autoencoder framework on user-item bipartite graphs to learn user and item embeddings. Many other works based on GNN [34,35] have been proposed in order to better capture the cooperative signals existing in user-item bipartite graphs. In order to make full use of other information besides user-item interaction, KGAT [36] constructed collaborative knowledge by combining user-item graph with knowledge graph and then applied graph convolution to obtain the final node representation. Although these GNN-based models have made progress, it is still challenging to apply them directly to CTR prediction. Fi-GNN [13] first introduced GNN to model feature interaction on the graph structure, but its simple aggregation method limited the ability to obtain cross features. Therefore, the work in this paper attempts to combine GNNs and attention mechanisms to model feature interactions of graph-structured features of multi-domain sparse data.

### 2.3. Attention Mechanism

Attention mechanisms originated in the field of neural machine translation [37] and have been successfully applied in many tasks, such as question answering systems [38], text summarization [39] and recommender systems [9,22,23]. The core idea of the attention mechanism is to learn to assign attention weights to a set of features to indicate the information contained in the corresponding features for the final task. In the field of recommender systems, DIN [2] designed a local activation unit to soft search for relevant user behaviors and employed weighted summation pooling to obtain an adaptive representation of user interests for a given advertisement. AFM [9] improves FM by distinguishing the importance of different feature interactions through a neural attention network. In this paper, we use an attention mechanism at the GNN interaction layer to select the intersecting features that contribute more to the model, resulting in better prediction performance.

## 3. The Proposed Method

**Figure 1:**
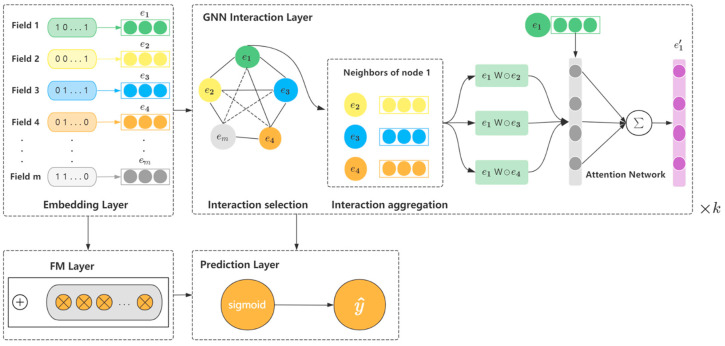
Diagram of the GAIAN model structure.

In this section, the GAIAN model proposed in this paper will be introduced, as shown in Figure 1. The model is mainly composed of the Embedding layer, FM layer, GNN feature interaction layer and prediction layer.

### 3.1. Embedding Layer

The input data of CTR prediction task usually consists of categorical features and numerical features. Since categorical features cannot directly participate in numerical computation, one-hot encoding is used to convert categorical features into binary vector representations. In general, input samples can be represented as:

(1) $$x=\left[x_{1},x_{2},\ldots ,x_{m}\right],$$

where m represents the number of feature fields, $x_{i}\in R^{n}$ represents a one-hot vector of a categorical feature domain with n features, $x_{j}\in R$ is a scalar whose numerical eigenfield has only one value. However, the one-hot encoding of categorical features is usually high-dimensional and sparse. If such high-dimensional and sparse feature vectors are directly input into deep neural networks for training, the computational complexity of the model will be too high and the generalization ability of the model will be reduced. A common approach to solve this problem is to introduce the Embedding layer. Specifically, a categorical feature $x_{i}$ is mapped to a dense embedding vector $e_{i}\in R^{d}$:

(2) $$e_{i}=V_{i}x_{i},$$

where $V_{i}$ is the embedding matrix of feature field i.

For a numerical feature $x_{j}$ whose scalar value is $x_{j}$ we also represent it in the d-dimensional embedding space:

(3) $$e_{j}=v_{j}x_{j},$$

where $v_{j}$ is the embedding vector of the numerical feature field j and $e_{j}$ is the embedding vector of the numerical feature $x_{j}$. Therefore, we can get a feature embedding matrix consisting of these feature embeddings:

(4) $$E_{m}={\left[e_{1},e_{2},\ldots ,e_{m}\right]}^{⊤},$$

### 3.2. FM Layer

Deep learning technology has outstanding performance in learning complex feature relations, but it is easy to ignore the relationship between simple features. In order to enable the model to learn a richer representation of feature relationships, improve the upper limit of the model’s decision-making ability, and speed up the fitting process, the model in this paper uses an FM [4] layer to learn simple relationships between features. In view of the high-dimensional and sparse features in the click-through rate prediction scene, the FM layer can quickly, efficiently and fully learn the low-order feature combination information, that is, the first-order and second-order feature combination information. The calculation method of FM layer output result is shown in Equation (5):

(5) $$y_{FM}=\;w_{0}+\;\overset{m}{\underset{i=1}{\sum }}w_{i}x_{i}+\frac{1}{2}\overset{d}{\underset{f=1}{\sum }}[{\left(\overset{m}{\underset{i=1}{\sum }}e_{i,f}x_{i}\right)}^{2}-\;\overset{m}{\underset{i=1}{\sum }}e_{i,f}^{2}x_{i}^{2}],$$

where $w_{0}$ represents the global bias, $w_{i}$ represents the weight of the ith feature, $x_{i}$ represents the input value of the ith feature, $e_{i,f}$ represents the element of vector $e_{i}$ is a concrete value, m represents the number of feature fields and $y_{\mathrm{FM}}$ represents the output of FM layer.

### 3.3. GNN Interaction Layer

**Figure 2:**
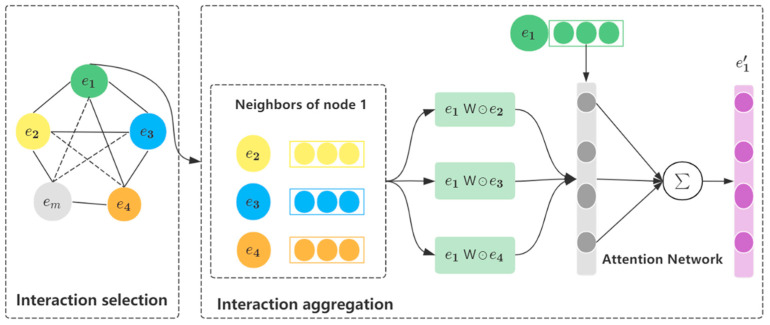
The GNN feature interaction layer.

The GNN interaction layer realizes k-order feature interaction by stacking k layers. The structure of each layer is shown in Figure 2, which mainly includes two steps of feature interaction selection and feature interaction aggregation.

#### 3.3.1. Feature Interaction Selection

Like Fi-GNN [13], the multi-field features of each input sample are represented as a feature map $G=\left(N,E\right)$, where each node $e_{i}\in N$ represents a feature field i, different feature fields can interact through edges. In contrast, considering that not all feature interactions are beneficial, we design an interaction selection mechanism in order to select beneficial feature interactions.

The adjacency matrix in the traditional GNN model is usually discrete, that is, only contains 0 and 1. It can only reflect the connection relationship between nodes, but cannot reflect the importance of the interaction between nodes. To overcome this problem, this paper replaces the edge set E with a weighted adjacency matrix A, where $A_{ij}$ represents the probability of edge $(e_{i},e_{j})\in E$ and also the importance of the interaction between node $e_{i}$ and node $e_{j}$. In particular, this paper learns a different graph structure representation $A^{\left(k\right)}$ at each layer. This is because if a fixed graph structure is used at each layer, then only a fixed set of feature interactions can be obtained, and by adaptively learning the graph structure at each layer, the model can model any potential feature interactions. The process of feature interaction selection is described in detail below.

**Metric Function.** To select beneficial feature interactions, we use an attention mechanism to learn edge weights to measure the importance of interactions between different nodes. In particular, the weights of the edges from node $e_{i}$ and node $e_{j}$ are computed using their initial node states, i.e., the corresponding feature embedding vectors. The formula is described as follows:

(6) $$a\left(e_{i},e_{j}\right)=\frac{\exp\left(LeakyReLU\left(W_{a}\left[e_{i}∥e_{j}\right]\right)\right)}{\sum_{k\in N_{i}}\;\exp\left(LeakyReLU\left(W_{a}\left[e_{i}∥\;e_{k}\right]\right)\right)},$$

where $W_{a}\in R^{2d}$ is a weight matrix, ∥ is the concatenation operation, and LeakyRelu is the activation function. To make the weights easy to compare across different nodes, we use the softmax function to normalize them. Therefore, the weighted adjacency matrix can be expressed as:

(7) $$A\left[e_{i},e_{j}\right]=\left\{\begin{array}{c} a\left(e_{i},e_{j}\right),\;\mathrm{if}\;i\neq j\\ 0,\;\mathrm{else} \end{array}\right.,$$

Since the weights of edges reflect the importance of different feature interactions, GAIAN can well explain the relationship between different feature fields in input samples.

**Graph Sampling.** For the weighted adjacency matrix $A^{\left(k\right)}$ of each layer, we sample the beneficial feature interactions, that is, the neighbor nodes of each feature field. In this paper, for each feature node we sample a fixed size neighborhood. For each feature node e of the kth layer, we select nk edges according to the n elements with larger weights in the weighted adjacency matrix *A*, the pseudocode is as follows:

(8) $$\begin{array}{c} \;\mathrm{for}\;i=1,2,\cdots ,m\\ \;\;\;\;\;\;\;\;index_{i}=argtop_{n_{k}}\left(A^{\left(k\right)}\left[i,:\right]\right)\\ \;\;\;\;\;\;\;\;A^{\left(k\right)}\left[i,-index_{i}\right]=0, \end{array},$$

where $A^{\left(k\right)}\left[i,:\right]$ represents the *i*-th row of the adjacency matrix $A^{\left(k\right)}$ in the *k*-th layer, $A^{\left(k\right)}\left[i,-index_{i}\right]$ represents the subset of the *i*-th row in the *k*-th layer that is not indexed by $index_{i}$. $argtop_{n_{k}}$ is an operator that selects $\;n_{k}$ element indices with larger weights from $A^{\left(k\right)}\left[i,:\right]$. We keep only $n_{k}$ feature nodes with larger weights, and the others are masked out. Therefore, the neighborhood set of node $e_{i}$ can be expressed as:

(9) $$N_{i}^{\left(k\right)}=\left\{e_{j}|a_{ij}^{\left(k\right)}>0,j=1,2,\cdots ,m\right\},$$

#### 3.3.2. Feature Interaction Aggregation

In a graph neural network, the traditional way to update the representation vector of each target node is to perform mean/sum/max pooling on the current target node embedding vector $h_{v}$ and its neighborhood information vector $h_{N\left(v\right)}$. However, these simple combinatorial methods limit the expressive power of graph neural networks because they cannot learn feature interactions between target node vectors and their neighborhood information vectors. To address this issue, this paper aggregates information from neighbor nodes using a bilinear interaction function.

**Figure 3:**
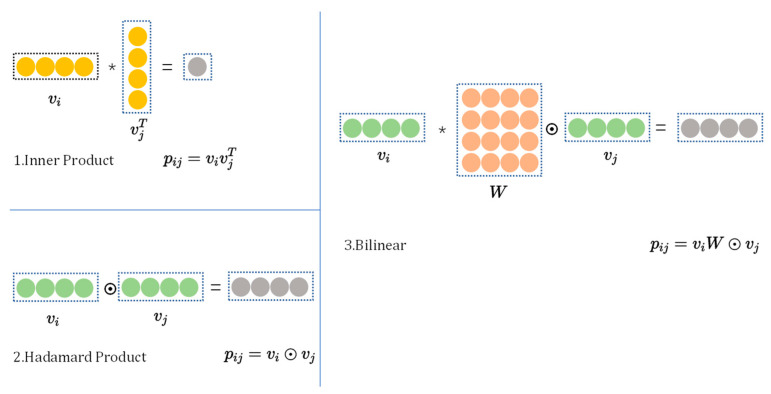
Three different types of interactions.

**Bilinear interaction function.** The classic methods of feature interaction are inner product and Hadamard product. Inner product is widely used in shallow models, such as FM [4] and FFM [5], while Hadamard product is often used in deep models, such as AFM [9] and NFM [11]. The forms of inner product and Hadamard product are expressed as $\left\{\left(e_{i}\cdot e_{j}\right)\right\},\left(i,j\right)\in R_{x}$ and $\left\{\left(e_{i}⊙e_{j}\right)\right\},\left(i,j\right)\in R_{x}$ respectively, where $R_{x}={\left\{\left(i,j\right)\right\}}_{i\in \left\{1,\cdots ,f\right\},j\in \left\{1,\cdots ,f\right\},j>i}$, $e_{i}$ is the embedding vector of the *i*-th feature field, ⋅ represents the inner product, ⊙ represents the Hadamard product. For example, $\left[a_{1},a_{2},a_{3}\right]⊙\left[b_{1},b_{2},b_{3}\right]=\left[a_{1}b_{1},a_{2}b_{2},a_{3}b_{3}\right]$. However, inner product and Hadamard product are too simple to effectively model feature interactions in sparse datasets [40]. Therefore, this paper uses a more fine-grained method that combines inner product and Hadamard product to learn feature interactions with extra parameters. As shown in Figure 3, the inner product is used between the matrix W and the vector $v_{i}$, and the Hadamard product is used between the matrix W and the vector $v_{j}$. Taking the embedding vector $e_{i}$ of the *i*-th feature field and the embedding vector $e_{j}$ of the *j*-th feature field as examples, the calculation method of the feature interaction $p_{ij}$ is as follows:

(10) $$p_{ij}=e_{i}W⊙e_{j},$$

where $W\in R^{d\times d}$ is shared among all $\left(e_{i},e_{j}\right)$ feature interaction pairs. $e_{i}$ and $e_{j}$ are the embedding vectors of the *i*-th and *j*-th feature domains, respectively, 1≤i≤f,i≤j≤f.

**Information Aggregation.** Most popular GNN models follow a neighborhood aggregation strategy, where node representation is achieved by iteratively aggregating the information of its neighbor nodes. Different cross features play different roles in the final prediction of the model. Therefore, for the target feature node $e_{i}$, when aggregating its feature interactions with neighbor nodes, this paper uses the attention network to calculate the attention coefficient of each interaction. The formula is described as follows:

(11) $$\begin{array}{c} c_{ij}^{'}=h^{T}LeakyReLU\left(W_{c}p_{ij}+b\right)\\ c_{ij}=\frac{\exp\left(c_{ij}^{'}\right)}{\sum_{j\in N_{i}^{\left(k\right)}\;}\exp\left(c_{ij}^{'}\right)} \end{array},$$

Among them, $W_{c}\in R^{t\times d}$, $b\in R^{t}$, $h\in R^{t}$ are the parameters of the model, and *t* represents the hidden layer size of the attention network. In order to make the attention coefficient easy to compare between different feature nodes, the attention coefficient $c_{ij}^{'}$ is then obtained by the softmax function to $c_{ij}$. After the attention coefficients are obtained, the updated representation of the target node is obtained by calculating the nonlinear combination of these feature interactions:

(12) $$e_{i}^{'}=\sigma \left(\underset{j\in N_{i}}{\sum }c_{ij}p_{ij}\right),$$

### 3.4. Prediction Layer

The output of the *k*-th layer of the GNN interaction layer is the representation vector $\left\{e_{1}^{\left(k\right)},e_{2}^{\left(k\right)},\ldots ,e_{m}^{\left(k\right)}\right\}$ of m feature nodes, which contains the k-order interaction information of the feature nodes. Since the interaction information of different orders of feature nodes is obtained in different layers, they have different contributions to the final prediction of the model. Therefore, we concatenate them to form the final representation of each feature:

(13) $$e_{i}^{*}=e_{i}^{\left(1\right)}∥\cdots ∥e_{i}^{\left(k\right)},$$

Finally, we average pool the vectors of all features to obtain the feature map level output and transform using the projection vector p to get the result of the GNN interaction layer:

(14) $$e^{*}=\frac{1}{m}\overset{m}{\underset{i=1}{\sum }}e_{i}^{*},$$

(15) $$y_{\mathrm{GNN}}=p^{⊤}e^{*},$$

The final prediction result is the addition of the results of the FM layer and the GNN interaction layer and then a value between 0 and 1 is obtained through a sigmoid function. The output result is calculated as shown in Equation (16).

(16) $$\hat{y}=sigmoid\left(y_{\mathrm{FM}}+y_{\mathrm{GNN}}\right),$$

### 3.5. Loss Function

In order to learn the parameters of the model, an objective function needs to be specified for optimization. Since CTR prediction is a binary classification task, logarithmic loss is used as the loss function, which is widely used for model training in previous work. The objective of the objective function is to minimize the cross-entropy of the predicted and true values, which is defined as:

(17) $$\mathrm{loss}=-\frac{1}{N}\overset{N}{\underset{i=1}{\sum }}\left(y_{i}\log\left({\hat{y}}_{i}\right)+\left(1-y_{i}\right)*\log\left(1-{\hat{y}}_{i}\right)\right),$$

where *N* represents the number of total samples, $y_{i}$ is the true value of the ith sample, and ${\hat{y}}_{i}$ is the model’s predicted value for the ith sample. Furthermore, the parameters are updated by minimizing the total loss using Adam [41].

## 4. Experiment

In this section, we conduct sufficient experiments to verify the following three questions:

**RQ1:** How does the proposed model perform compared to the current state-of-the-art CTR prediction methods?

**RQ2:** Does the GAIAN model perform better than the original GNN in modeling higher-order feature interactions?

**RQ3:** How does the setting of the model parameters affect the performance of the GAIAN model?

We first introduce some basic experimental settings in detail and then answer the above questions.

### 4.1. Experiment Setup

#### 4.1.1. DataSet

To evaluate the effectiveness of the GAIAN model, this paper conducts a series of experiments on two well-known public datasets. The statistics of these datasets are summarized in Table 1.

**Criteo** is a well-known real-world display advertising dataset with impression information and corresponding user click logs for each ad. This dataset is widely used in the evaluation of various CTR prediction models. It contains user click logs for 45 million samples each of which has 39 features, including 13 continuous numerical features and 26 categorical features.

**Avazu** dataset includes a record of user clicks on mobile ads for several consecutive days and 40 million data samples. This dataset was originally used for the Kaggle CTR prediction competition and has since been widely used as an evaluation benchmark for many CTR prediction models.

For the two datasets, first, we remove the infrequent features that occur less than 10 and 5 times respectively, and treat them as a single feature “<unknown>”. Second, since numerical features may have large variance, we normalize numerical values by transforming a value *z* to $\log^{2}\left(z\right)$ if *z* > 2. Finally, we randomly select 80% of all samples for training and randomly split the rest into validation and test sets of equal size.

#### 4.1.2. Evaluation Metrics

This paper uses the following two metrics for model evaluation: AUC (area under the ROC curve) and Logloss (cross entropy).

**AUC**: The area under ROC curve is usually used to judge the performance of binary prediction model. The lower limit of AUC is 0 and the upper limit is 1. The higher the value, the better the performance of the model.

**LogLoss**: LogLoss is a widely used measurement method in binary classification problems. It is used to measure the distance between two distributions, that is, the gap between the true value and the predicted value. The lower bound of logarithmic loss is 0, which means that the two distributions match exactly. The smaller the value, the better the performance.

It is worth noting that a slightly higher AUC or lower LogLoss at the 0.001 level is significant for the CTR prediction task, which has also been pointed out in existing work.

#### 4.1.3. Baseline Methods

This paper compares the GAIAN model with three types of existing methods: (1) a linear combination of original features to model first-order interactions; (2) an FM-based approach to model second-order feature interactions; and (3) Models for modeling higher-order feature interactions based on deep learning. These methods are briefly described below:

- **LR (Logistic Regression)** [42] captures first-order cross features by linearly combining the original features;
- **FM** [4] uses the inner product of feature vectors to model second-order feature interactions;
- **AFM** [9] uses attention mechanism on the basis of FM to distinguish the importance of different second-order crossover features;
- **Deep & Cross** [43] try to model feature interactions in an explicit way by taking the outer product of feature vectors at the bit-wise level;
- **xDeepFM** [44] uses a compressed interaction network to explicitly capture finite-order feature interactions;
- **AutoInt** [22] utilizes a multi-head self-attention neural network with residual connections to model higher-order feature interactions in an explicit manner;
- **Fi-GNN** [13] models features as a fully connected graph and utilizes a gated graph neural network to model higher-order feature interactions;
- **AFN** [45] designed a Logarithmic Transformation Layer to automatically capture feature interactions of any order.

#### 4.1.4. Implementation Details

This article uses Tensorflow to implement all the models. For the Embedding layer, the embedding dimension is set to 8 for the Criteo dataset and 32 for the Avazu dataset. For the optimization method, we use Adam [41], the batch-size of the Criteo dataset is 1024, the batch-size of the Avazu dataset is 512, and the learning rate is set to 0.001. For all deep models, the layer depth is set to 3, all activation functions are ReLU; the number of neurons per layer is 256 for the Criteo dataset, 512 for the Avazu dataset, and the dropout is set to 0.5.

### 4.2. Performance Comparison (RQ1)

According to the experimental setup, this paper compares the GAIAN model with various models, including models with first-order, second-order, and higher-order feature interactions. The performance comparison of these methods on the two datasets is shown in Table 2 from which the following observations are made:

(1) Compared with other nonlinear models, the linear model LR fails to achieve good performance on both datasets, which indicates that modeling the nonlinear relationship between features is crucial to improve the accuracy of CTR prediction;
(2) FM and AFM capture second-order nonlinear feature interactions and achieve better prediction results compared to LR, which indicates that pairwise interactions through the inner product of feature vectors have a positive impact on prediction performance. Furthermore, AFM outperforms FM on both the Criteo and Avazu datasets, suggesting that the importance of distinguishing different feature interactions enables the model to have better predictive performance;
(3) On the Criteo and Avazu datasets, most higher-order models outperform models that learn only low-order feature interactions, suggesting that modeling higher-order interactions among features is more important for predictive performance;
(4) Compared with other baseline models, Fi-GNN has better prediction performance, indicating that the feature interaction based on graph neural network has stronger feature representation ability;
(5) The GAIAN model yields the best performance on both the Criteo and Avazu datasets and achieves innovative improvements over state-of-the-art models. On the Criteo and Avazu datasets, GAIAN has the highest AUC values (0.8011 and 0.7769) and the lowest LogLoss values (0.4588 and 0.3871). It is worth noting that a slightly higher AUC or a lower LogLoss at the 0.001 level is considered a significant improvement in the CTR prediction task, which has also been pointed out in existing work [21,22]. Due to the effectiveness of integrating graph structure and attention mechanism to model feature interactions, the proposed model has great advantages over the most advanced models.

### 4.3. Ablation Study (RQ2)

In order to verify the effectiveness of the individual components of the GAIAN model, this section compares the different variants of the GAIAN model by eliminating the different key components in the GAIAN model. Variants of these GAIAN models are defined as follows:

- GAIAN: The complete GAIAN model;
- GAIAN-1: Feature interaction selection is the first component of each layer of GAIAN, which selects only beneficial feature interactions. To verify the necessity of this component, this component is removed so that all pairwise feature interactions are modeled as a fully connected graph;
- GAIAN-2: In the feature interaction aggregation component, this paper aggregates the feature interactions between the central node and the neighbor nodes, instead of aggregating the neighbor features as in the standard GNN. In order to verify its rationality, this paper tests the performance of the model when the neighborhood features are directly aggregated. We use a single type of aggregator to obtain neighborhood characteristics in GAIAN-2, GAIAN-3, and GAIAN-4. In GAIAN-2, we only use an average pooling aggregator to obtain information about neighbor nodes;
- GAIAN-3: In this variant, the aggregation stage uses a max-pooling aggregator;
- GAIAN-4: In this variant, the aggregation stage uses an attention mechanism as the aggregator.

**Figure 4:**
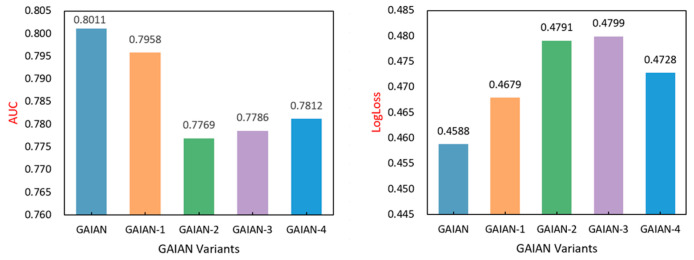
The performance of different variants of GAIAN on the Criteo dataset.

**Figure 5:**
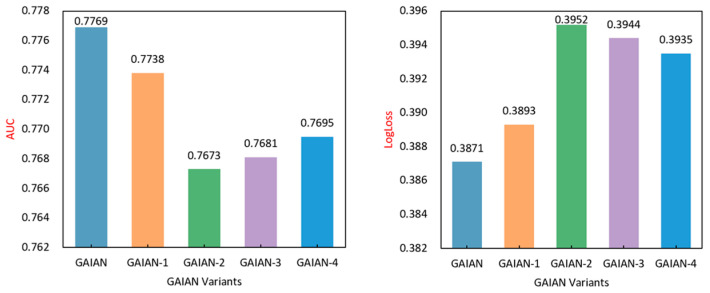
The performance of different variants of GAIAN on the Avazu dataset.

The experimental results are shown in Figure 4 and Figure 5. The full GAIAN model outperforms other variants and verifies that each component has its contribution. GAIAN-1 verifies the effectiveness of the feature interaction selection component. Through the feature interaction selection component, we select cross features that are beneficial to model prediction, reduce model noise and reduce the risk of model overfitting. GAIAN-2, GAIAN-3 and GAIAN-4 verify the effectiveness of the feature interaction aggregation component. Compared with the original GNN model, this paper extracts fine-grained intersection features in a flexible and explicit way by integrating the bilinear interaction function into the aggregation strategy of the graph neural network, thus improving the model’s ability to capture user preferences. It also shows that explicit feature interaction helps to improve the expressiveness of graph nodes.

### 4.4. Parameter Study (RQ3)

In this subsection, some hyperparameters of the model will be investigated. We mainly focus on the hyperparameters of the following parts in the GAIAN model: (1) the dimension of embedding; (2) the order of feature interaction.

#### 4.4.1. Dimensions of the Embedding Section

Changes in the embedding dimension will affect the number of model parameters. The experiment compares the impact of different embedding dimensions on the prediction performance of GAIAN, hoping to obtain a suitable embedding dimension to obtain better model performance. In this paper, the size of the embedding dimension is gradually adjusted from 8 to 64, and the experimental results are shown in Table 3.

From the experimental results, it can be observed that:

- On the Avazu dataset, when the dimension of the embedding vector is lower than 32, the performance of the model continues to increase, because the larger embedding dimension can retain more information. When the dimension of the embedding vector exceeds 32, the model performance begins to decline. This is because the increase of the embedding dimension means an increase in the number of model parameters, which easily leads to overfitting of the model;
- Performance degrades when increasing the size of the embedding vector dimension on the Criteo dataset. This may be because the Criteo dataset has more features than the Avazu dataset. When the dimension of the embedding vector is increased, too many model parameters lead to optimization difficulties.

#### 4.4.2. Order of Feature Interactions

**Figure 6:**
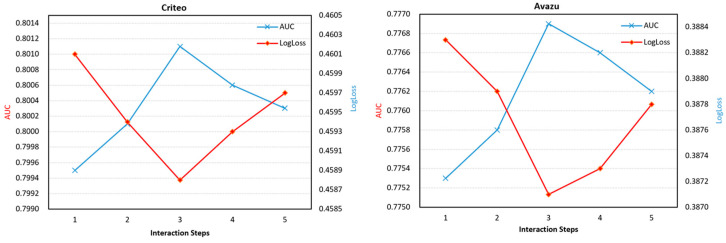
Performance of GAIAN model under different feature interaction orders.

In the graph structure, the order of feature interactions is equal to the number of iterations of the graph neural network. In order to explore the influence of different feature interaction orders on the model performance, this section studies the performance of the GAIAN model under different iterations, and the experimental results are shown in Figure 6.

An appropriate number of iterations can effectively improve the predictive performance of the model. According to the experimental results, it can be observed that for the Criteo and Avazu datasets, when the number of iterations is set to 3, the model proposed in this paper can achieve the best performance. This is because the Criteo and Avazu datasets have 39 and 23 feature domains, respectively, and 3 iterative steps are sufficient to generate higher-order feature interactions.

## 5. Conclusions and Future Work

This paper is aimed at the over-fitting problem of the model caused by too much noise due to the lack of processing of invalid features in the previous model. At the same time, in order to give full play to the advantages of GNN in explicitly modeling, higher-order feature interactions, a CTR prediction model GAIAN based on graph attention interaction aggregation network is proposed, which can explicitly and automatically learn higher-order feature interactions. The key to our approach is to design a feature interaction selection mechanism using a hard attention mechanism, reducing the risk of model overfitting. At the same time, the bilinear interaction function is integrated into the aggregation strategy of the graph neural network, which improves the interpretability of the feature interaction. Extensive experiments on multiple real datasets demonstrate that the proposed model has better offline AUC and Logloss values than other state-of-the-art models. However, the model proposed in this paper also has certain shortcomings. In the future, we will enhance the robustness of the model and explore methods for more efficient feature interaction modeling to improve the performance of the CTR prediction model.

## Figures and Tables

**Table 1:** Dataset Statistics.

Dataset	Instances	Fields	Feature
Criteo	45,840,617	39	998,960
Avazu	40,428,967	23	1,544,488

**Table 2:** Performance comparison of different models.

Model Type	Model	Criteo		Avazu	
		AUC	LogLoss	AUC	LogLoss
First-order	LR	0.7653	0.4877	0.7393	0.4146
Second-order	FM	0.7769	0.4775	0.7509	0.4044
	AFM	0.7818	0.4724	0.7587	0.4031
High-order	Deep&Cross	0.7938	0.4681	0.7679	0.3956
	xDeepFM	0.7929	0.4686	0.7671	0.3949
	AutoInt	0.7993	0.4609	0.7754	0.3887
	Fi-GNN	0.7996	0.4604	0.7758	0.3889
	AFN	0.7983	0.4621	0.7731	0.3903
	GAIAN	0.8011	0.4588	0.7769	0.3871

**Table 3:** Performance comparison of different models.

Embedding Size	Criteo		Avazu	
	AUC	LogLoss	AUC	LogLoss
8	**0.8011**	**0.4588**	0.7752	0.3898
16	0.8004	0.4597	0.7757	0.3895
32	0.8001	0.4601	**0.7769**	**0.3871**
64	0.7996	0.4609	0.7761	0.3890

## Data Availability

The Criteo dataset supporting this study was obtained from https://www.kaggle.com/c/criteo-display-ad-challenge/data, accessed on 10 September 2022. The Avazu dataset was obtained from https://www.kaggle.com/c/avazu-ctr-prediction/data, accessed on 18 September 2022.
